# Low Duty-Cycling MAC Protocol for Low Data-Rate Medical Wireless Body Area Networks

**DOI:** 10.3390/s17051134

**Published:** 2017-05-16

**Authors:** Chongqing Zhang, Yinglong Wang, Yongquan Liang, Minglei Shu, Jinquan Zhang, Lina Ni

**Affiliations:** 1 College of Computer Science and Engineering, Shandong University of Science and Technology, Qingdao 266590, China; zhangchongqing@sdust.edu.cn (C.Z.); lyq@sdust.edu.cn (Y.L.); tjzhangjinquan@126.com (J.Z.); 2 Shandong Provincial Key Laboratory of Computer Networks, Shandong Computer Science Center, National Supercomputer Center in Jinan, Jinan 250101, China; wangyl@sdas.org (Y.W.); shuml@sdas.org (M.S.); 3 The Key Laboratory of Embedded System and Service Computing, Ministry of Education, Tongji University, Shanghai 200092, China

**Keywords:** wireless body area networks (WBANs), energy-efficient, MAC protocol, low data-rate, low duty-cycling

## Abstract

Wireless body area networks (WBANs) are severely energy constrained, and how to improve the energy efficiency so as to prolong the network lifetime as long as possible is one of the most important goals of WBAN research. Low data-rate WBANs are promising to cut down the energy consumption and extend the network lifetime. Considering the characteristics and demands of low data-rate WBANs, a low duty-cycling medium access control (MAC) protocol is specially designed for this kind of WBAN in this paper. Longer superframes are exploited to cut down the energy consumed on the transmissions and receptions of redundant beacon frames. Insertion time slots are embedded into the inactive part of a superframe to deliver the frames and satisfy the quality of service (QoS) requirements. The number of the data subsections in an insertion time slot can be adaptively adjusted so as to accommodate low data-rate WBANs with different traffic. Simulation results show that the proposed MAC protocol performs well under the condition of low data-rate monitoring traffic.

## 1. Introduction

The rapid rises of the aging population and healthcare costs have been two major challenges that modern societies have to face. Taking the USA as an example, the number of people aged over 60 in 2050 is expected to be 81 million, which is about 2.5-times that of the year 2000 (33 million). Elderly people are vulnerable to many chronic diseases, e.g., cardiovascular disease, Parkinson’s disease, asthma and diabetes. This will lead to a rapid increase of healthcare expenditure, which will overload the healthcare system and significantly lower the life quality. All of these necessitate reforms of the current healthcare system and call for innovative solutions to healthcare problems [1].

The resultant force from the advances of microelectronics, wireless communications, intelligent sensors and batteries has given rise to the birth of wireless body area networks (WBANs), which are hoped to launch a wave of innovative applications in medical, entertainment, gaming, sports and fitness fields. WBANs are promising to create a revolutionary healthcare model, which can greatly improve the patients’ life quality. In addition, WBANs can provide proactive wellness management by focusing on early detection and prevention of diseases [2].

A medical monitoring WBAN usually takes on a star structure, which is composed of one coordinator and some sensor/actuator nodes. WBANs can be regarded as a special type of wireless sensor network (WSN) [3]. Like a WSN node, a WBAN node generally is also powered by batteries. It is inconvenient and even infeasible to replace or recharge these batteries. Therefore, the WBAN nodes are severely energy constrained, especially for the WBAN nodes implanted in the human body. As a result, how to reduce the energy consumption of the nodes so as to prolong the lifetime of the nodes and even the network lifetime is one of the most important goals of WBAN research [4]. For a WBAN node, the most energy-consuming part is the antenna, and the most energy-consuming operations are transmissions and receptions of data. To extend the lifetime of a node as much as possible (e.g., from days to months or even years), the only way is to greatly reduce the duty cycle of the antenna [5].

A WBAN node may adopt two kinds of monitoring modes, periodic mode and random mode [6]. Some periodic monitoring applications, e.g., electrocardiogram (ECG), electroencephalogram (EEG) and electromyogram (EMG), may generate quite high traffic, which may result in a high duty cycle and cause high energy consumption. Comparatively speaking, other periodic monitoring applications, e.g., body temperature and blood saturation, and random monitoring applications only bring about low traffic, which only incurs low energy consumption. Hence, to reduce the duty cycle effectively, it is necessary to reduce the traffic incurred by the high data-rate periodic monitoring applications. By introducing in-network processing [7,8], the data can be processed and judged on the nodes, and the data are transmitted to the coordinator only when needed. For example, by processing and judging the data on the node, a cerebral hemorrhage monitoring node only needs to send the data to the coordinator when certain events are detected. Such techniques can greatly reduce the traffic of a high data-rate periodic monitoring node and realize a new kind of low data-rate monitoring mode. Furthermore, such techniques can transform high data-rate WBANs to be a new kind of low data-rate WBANs [9].

The traffic patterns generated by a high data-rate WBAN and a low data-rate WBAN are quite different. Generally speaking, nodes working in the high data-rate monitoring mode produce busy periodic traffic, which consumes a mass of communication resource. Transporting these vast quantities of data from the nodes to the coordinator means a high cost of energy because communication dominates the energy consumption under such circumstances [10]. As a consequence, the lifetime of a node adopting this monitoring mode will be severely shortened. Compared to a high data-rate WBAN, the traffic generated by a low data-rate WBAN may be rather sporadic and low. Hence, the prominent advantages of the low data-rate WBANs lie in the greatly reduced communication energy, which means a much longer service time [9].

High data-rate WBANs and low data-rate WBANs have their own advantages and usages. A high data-rate WBAN can collect vast quantities of detailed information about one patient’s body in a long period of time so that the medical staff can master the change-rules through analyzing this information. This undoubtedly can help the medical staff closely trace or build a deep perception of the patient’s health status. Such a WBAN is suitable for patients whose illness condition is severe or unstable, or people who need long-term information to build their health models [11]. Compared to the high data-rate monitoring mode, a low data-rate WBAN only delivers data to the coordinator when some abnormal conditions are detected. As a result, it cannot provide enough detailed information for the medical staff to trace the patient’s health in the long run in detail, but it still can afford proactive wellness management, including early detection and prevention of diseases. Such a WBAN is suitable for patients that do not have a bad health condition, or that have a stable illness condition, or people who have built their health models.

High data-rate WBANs and low data-rate WBANs do not contradict each other. On the contrary, they complement each other. When a patient is in good conditions, all nodes of the WBAN can work in the low data-rate monitoring mode to save energy. When an emergency is detected, all nodes or a part of them can be switched into the high data-rate monitoring mode so as to obtain detailed information of the patient. By utilizing these two modes in a combined way, the quality of service (QoS) demands of a WBAN can be guaranteed, on the one hand, and the network lifetime can be prolonged on the other hand. In other words, in order to extend the network lifetime, a WBAN should stay in low data-rate monitoring mode as much as possible. This is hoped to provide a meaningful and potential energy management method for severely energy-constrained WBANs [9].

Medium access control (MAC) protocols are one key supporting technique of WBANs. MAC protocols have a significant impact on the performance of a WBAN by coordinating the nodes to access the medium and operating the most energy-consumed part, i.e., the antenna. With the help of an adequate MAC protocol, the duty cycle of a low data-rate WBAN node can be compressed effectively to reduce the energy consumption, which means a significantly extended network lifetime [12].

In this paper, we try to design a MAC protocol for low data-rate WBANs. One basic characteristic of a low data-rate WBAN is its low traffic. The proposed MAC protocol tries to take full advantage of this characteristic to reduce the duty cycle of a node to a very low level (thus, it is called low duty cycling MAC (LDC-MAC)). By extending the length of the superframe, LDC-MAC can reduce the number of beacon frames to cut down the overhead of transmitting and receiving redundant frames caused by a short superframe structure, which is adopted by the existing WBAN MAC protocols. To meet the real-time demands of time-critical traffic, short insertion time slots are embedded into the superframe. The number and length of the insertion time slots can be adjusted to adapt to the traffic fluctuation. Besides, LDC-MAC has several mechanisms to awaken the nodes to high data-rate monitoring mode.

The rest of this paper is organized as follows. The related works are introduced in the next section. The third section presents LDC-MAC’s superframe structure and how LDC-MAC operates to deliver different types of data. The performance of LDC-MAC is analyzed and evaluated in the forth section and the fifth section, respectively. Finally, we draw conclusions in the sixth section.

## 2. Related Works

Many MAC protocols, e.g., DQBAN (Distributed Queuing Body Area Network protocol) [13], EELDC-MAC (Energy-efficient Low Duty Cycle MAC protocol) [14], Med-MAC (Medical MAC protocol) [15], BodyMAC [16], TDMA-based-MAC [17], IEEE 802.15.4 [18], IEEE 802.15.6 [19], priority-based MAC [20], on-demand MAC [21], etc., have been proposed to support WBANs. In general, these MAC protocols take the characteristics of all kinds of traffic into consideration and exert two kinds of MAC mechanisms, CSMA/CA (carrier sense multiple access with collision avoidance) [22] and TDMA (time division multiple access) [23], to adapt to all sorts of traffic. Both mechanisms have their advantages and drawbacks. TDMA outscores CSMA/CA at energy efficiency, the utilization of bandwidth and network throughput. However, TDMA has its own shortcomings rooted in its inadaptability to the network changes and its dependency on the network time co-gradient [24]. Researchers have reached an agreement that TDMA is more suitable for WBANs with high periodic traffic and infrequent network changes, while CSMA/CA is more effective for WBANs with low traffic and frequent network changes [25,26].

In order to fulfill the different demands of periodic traffic and random traffic, many hybrid MAC protocols [27,28] that integrate the advantages of CSMA/CA and TDMA have been put forward. 802.15.4 and 802.15.6 belong to hybrid MAC protocols, as well. Generally speaking, TDMA is exploited by these protocols to handle busy traffic to avoid collisions and cut down the energy consumption, and CSMA/CA is adopted to deal with traffic incurred by random events and network commands such as time slot request, time slot release, node associate request, node disassociated release, etc. [29].

**Figure 1:**
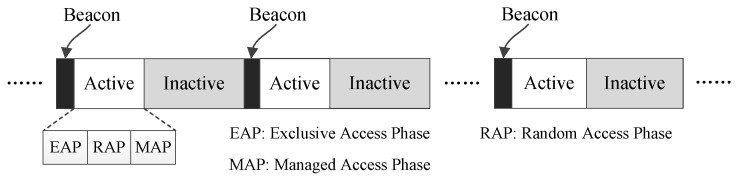
The scenario that 802.15.6 is applied to a low data-rate WBAN.

In Figure 1, 802.15.6 is taken as an example to illustrate the scenario when a hybrid MAC protocol is applied to a low data-rate WBAN. As the figure shows, a superframe of 802.15.6 consists of three periods, a beacon period, an active period and an inactive period. The active period may consist of some optional phases such as an exclusive access phase (EAP), a random access phase (RAP) and a managed access phase (MAP). EAP phases are mainly used to serve traffic of high priority such as the data generated by critical events, and RAP phases are used to serve traffic of common priority. Both EAP phases and RAP phases adopt the CSMA/CA mechanism. An MAP phase may use TDMA to support access modes like polling and scheduled allocation [30]. From this, it can be seen that CSMA/CA and TDMA can be exploited by 802.15.6 in a combined way to obtain higher medium access efficiency.

However, there are several drawbacks when 802.15.6 is applied to a low data-rate WBAN. For one thing, it is necessary to employ a short superframe structure to meet the real-time demand of time-critical traffic. This is bound to introduce unnecessary beacon frames, which lead to energy waste. Moreover, allocating how many time slots for EAP, RAP and MAP also has a big influence on the network performance. A long time length may cause communication resource waste, while a short time length may lead to long delays and even frame losses. Besides, it is necessary to design a fast wakeup mechanism for low data-rate WBANs. By such a mechanism, the low data-rate monitoring nodes can be awakened rapidly to high data-rate monitoring mode to collect detailed information when certain events are detected. Such a mechanism can also be used to drive an actuator to perform some actions, e.g., inject a certain dose of insulin into the body.

Time-slotted channel hopping (TSCH) has been introduced by the IEEE 802.15.4e standard [31] as one of its medium access control modes. By combining time-slotted access and channel hopping together, TSCH can provide predictable latency, high energy efficiency, reliable communication and high network capacity. LDC-MAC is similar to TSCH to some extent. There are dedicated links and shared links in TSCH, and LDC-MAC has similar concepts. The main difference between TSCH and LDC-MAC lies in the following aspects. Firstly, TSCH needs the support of multiple channels, while LDC-MAC works with only one channel. Secondly, TSCH is suitable for wireless sensor and actuator networks (WSANs) working in harsh conditions, e.g., industrial or intra-vehicle environments [32], while LDC-MAC is designed for low data-rate medical monitoring WBANs. Thirdly, CSMA/CA is used for shared links in TSCH, and Aloha [33] is adopted in LDC-MAC.

Besides the above protocols, many other MAC protocols have been proposed specially for handling emergency events. As an effective technique to awaken sleeping nodes, a wake up radio has been exploited by some MAC protocols [21,34,35]. The main drawbacks of this mechanism lie in the increase of the node cost and the node size. Some other MAC protocols have been designed without the help of the wake up radio [36,37]. For these protocols, the real-time requirements are generally satisfied by inserting additional time slots into the superframe. There are several shortcomings concerning these protocols. Firstly, these protocols are not suitable for accommodating a WBAN with a large number of nodes. Secondly, these protocols did not consider the requirements of actuator nodes. Moreover, these protocols failed to make full use of the characteristics of low data-rate monitoring mode to design energy-efficient mechanisms.

In [38], the authors have designed an energy-efficient WBAN MAC protocol named MEM-MAC (Medical Emergency Monitoring MAC protocol) for monitoring medical emergencies. As the medical emergency traffic is fairly low, MEM-MAC can effectively cut down the node energy consumption effectively and satisfies the delivery delay at the same time. However, as the medical emergency traffic becomes high, the performance of MEM-MAC deteriorates very quickly. The reason to explain this is that only one DATA subsection is installed in an interposition time slot, and two contention access periods (CAP) are exploited to handle the collisions and big data. Moreover, the superframe of MEM-MAC is a fixed structure, which makes it so that MEM-MAC only can accommodate a medical emergency monitoring WBAN in which there are no high data-rate monitoring nodes.

## 3. MAC Protocol Design

### 3.1. The Superframe Structure

A hybrid WBAN MAC protocol generally has two operation modes, i.e., beacon-enabled mode and non-beacon mode. In beacon mode, communications are controlled by the coordinator, which transmits beacons for time synchronization and network control. One prominent advantage of this mode is that the coordinator can manage and regulate the superframe structure elaborately. Another advantage of beacon mode is that the coordinator can reach the nodes conveniently, and this implies that it is easy to implement unicast or broadcast from the coordinator to the node using this mode. In beacon mode, the superframe may consist of an active phase and an inactive phase. The communications are carried out during the active period. To save energy, it would be best to set the inactive part to be as long as possible. However, this impairs the real-time performance. To ensure the real-time demands, the length of the superframe cannot be too long. Additionally, this signifies frequent transmitting and receiving of beacons, which may cause nontrivial energy waste [39].

In non-beacon mode, a node does not need to wake up periodically to get the beacon frames. Therefore, this mode can eliminate the energy waste caused by transmitting and receiving of beacons. The disadvantage of this mode lies in the inability of the coordinator to access the nodes freely. To communicate with the nodes, the coordinator must poll the nodes, or wait to be polled by the nodes. Using this mode, it is hard to wake up the nodes or drive an actuator to perform its actions. Under this mode, a node can send data to the coordinator at any time. This can guarantee low delivery delay from the nodes to the coordinator. Yet in order to receive data from the nodes, the coordinator needs to stay in listening mode as long as it does not need to transmit data. This apparently sacrifices the energy-efficiency of the coordinator [39].

From the above analysis, although non-beacon mode can realize high energy-efficiency for the nodes and low delay communication from nodes to the coordinator, it is hard to realize low delay communications from the coordinator to nodes and a rapid wake up mechanism. Besides, the coordinator suffers from low energy efficiency in this mode. Therefore, non-beacon mode is not adopted in the design of LDC-MAC. Compared to non-beacon mode, the disadvantages of beacon mode are the energy wasted on beacons and the probable long delivery delay from the nodes to the coordinator. By taking the advantages of beacon mode and designing effective mechanisms to overcome its disadvantages, LDC-MAC can satisfy the demands of nodes working in low data-rate monitoring mode.

**Figure 2:**
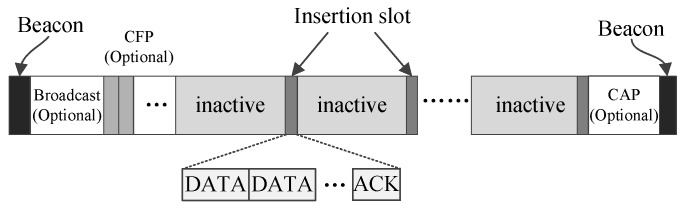
The basic superframe structure of low duty cycling MAC (LDC-MAC).

Figure 2 shows LDC-MAC’s basic superframe structure, which is used when all nodes of a WBAN are working in low data-rate monitoring mode. The superframe starts with a beacon frame that holds control information. Following the beacon frame, there may be a broadcast period and a contention-free period (CFP), which contains some guaranteed time slots (GTS). These two periods are both optional. The lengths of the broadcast period and the CFP period are included in the beacon frame. The coordinator uses the broadcast period to transmit long frames to the nodes. The GTS time slots in the CFP period are allocated to different nodes to transmit long frames. Whether these two optional periods exist or not relies on the foregoing superframe. If the coordinator generates big broadcast data or receives such data from an outside network during a superframe, then the coordinator stops the current superframe and begins a new superframe in which the optional broadcast period is enabled and exploited to broadcast the long data frame. If there are big data generated by node *N* in the course of the current superframe and node *N* judges that some GTS time slots are needed to transmit the big data, node *N* will send a GTS request to the coordinator for further handling of these data. Next, the coordinator will terminate the current superframe and commence a new one in which some GTS slots are allocated for node *N* to transmit its data.

The following phase is a long inactive phase that occupies most of the superframe. Because the traffic is low when all nodes are working in low data-rate monitoring mode, a long inactive period can help the coordinator and the nodes to save energy. To meet the real-time demands of the traffic, insertion time slots are inserted into the inactive phase to deliver data. Insertion time slots will be introduced in detail later. At the end of the superframe, there may be an optional CAP period, which can be shared by the nodes with data to deliver using CSMA/CA. Such a CAP period will be activated when frame collisions happen in one superframe.

**Figure 3:**
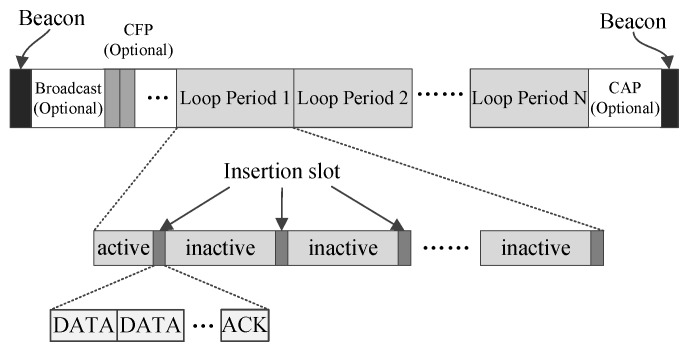
The superframe structure of LDC-MAC for supporting a WBAN in which low data-rate nodes and high data-rate nodes coexist.

The superframe structure displayed in Figure 2 is used to accommodate a WBAN in which all nodes are working in low data-rate monitoring mode, and it cannot satisfy the demands of a WBAN in which there are nodes working in high data-rate monitoring mode. The superframe structure shown in Figure 3 is designed for such occasions. In Figure 3, loop periods replace the inactive period in Figure 2. One loop period begins with an active period that is used for the nodes working in high data-rate monitoring mode to transmit their data. The active period can have a complex structure to accommodate the nodes working in high data-rate monitoring mode. The rest of one loop period is similar to the inactive part of Figure 2, which contains a long inactive period and many inserted insertion slots.

From Figure 2 and Figure 3, it can be observed that one insertion slot is composed of one data (DATA) section and one acknowledgement (ACK) section, and the DATA section can further contains multiple DATA subsections. The DATA subsections are used to convey data for the coordinator and the nodes working in the low data-rate monitoring mode. If the low data-rate monitoring traffic in a WBAN is rather low, then only one DATA subsection can be configured in one insertion time slot. In such a case, LDC-MAC will become very similar to MEM-MAC in which only one DATA subsection is installed in the interposition time slot. In fact, MEM-MAC can be viewed as one special case of LDC-MAC, or LDC-MAC can be deemed as the generalization of MEM-MAC. The transportation ability of only one DATA subsection is very limited and only can serve sparse traffic. LDC-MAC is designed to handle low data-rate monitoring traffic that is busier than the medical emergency traffic that MEM-MAC is supposed to handle. For this, LDC-MAC is redesigned to have the ability of installing multiple DATA subsections in one insertion time slot. One DATA subsection can be monopolized by one node or can be shared by multiple nodes. On the occasions that low data-rate monitoring traffic becomes high, the number of DATA subsections can be increased to handle the increasing traffic. As the traffic goes down, the number of DATA subsections can be axed to reduce the length of the insertion time slot. Because the traffic generated by the low data-rate monitoring nodes is generally low, so the collision possibility in a DATA subsection is also low. Based on this, Aloha [33] is adopted by the nodes for transmitting their frames in the DATA subsections.

In a WBAN, data communications are in two directions, from nodes to the coordinator and from the coordinator to nodes. Correspondingly, two types of DATA subsections are designed. The first type of DATA subsections is called the upload subsections, and the second type of DATA subsections is called the download subsections. In this paper, an insertion time slot only contains upload DATA subsections is called an upload insertion time slot. An insertion time slot only contains download DATA subsections is called a download insertion time slot. An insertion time slot that contains both upload DATA subsections and download DATA subsections is called a bidirectional insertion time slot.

To save energy, only one ACK section is installed in one insertion time slot for the coordinator to acknowledge all nodes. If there are multiple DATA subsections existing in an insertion time slot, the ACK section is used for the coordinator to acknowledge all potential nodes. This means the ACK frame, which contains all of the acknowledgment information, is broadcast to all of the nodes during the ACK section. No ACK section is designed for the nodes to acknowledge the coordinator. The reason is that the coordinator can only be acknowledged with one ACK section when it unicasts one frame to one node, while multicast and broadcast cannot be acknowledged easily and must be acknowledged with many ACK subsections. Based on this, there is only one case that the coordinator can be acknowledged, that is, only one download DATA subsection, and no upload DATA subsection is included in an insertion slot.

The download DATA subsections and the ACK sections can also be adopted by the coordinator to issue control frames. The control frames can be used to break a superframe and begin a new one, broadcast synchronization clock information, commence a CAP period, etc. In a superframe, all intervals between adjacent insertion slots have a fixed length. Different superframes can have different insertion intervals. The lengths of the intervals are mainly set according to the real-time demands. Besides, the insertion intervals also depend on the occurrence of low data-rate monitoring traffic.

**Figure 4:**
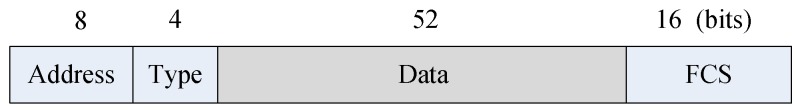
The structure of the insertion DATA frame.

Figure 4 illustrates the frame structure transmitted in one DATA subsection of an insertion time slot. The total length of such a frame is 10 bytes, which are divided into four fields. It is worth noting that the data field length is only 52 bits, which is only enough for ordinary commands and data. Such data are called small data. The data that cannot be delivered directly by such a DATA frame are called big data. To deliver it, the sender node creates a GTS request and transmits it to the coordinator. A priority class value is included in the request, and the coordinator judges this value and allocates GTS time slots in the following superframe. Big data are always transmitted using the GTS slots in the next new superframe.

**Figure 5:**
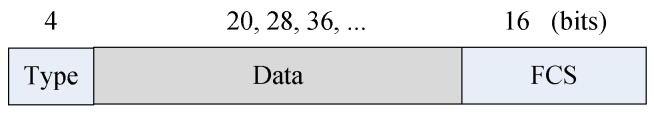
The structure of the insertion ACK frame.

Figure 5 shows the frame structure transmitted in the ACK section. ACK frames are mainly used by the coordinator to acknowledge DATA frames from nodes. The length and structure of the ACK frame vary with the number of DATA subsections and the number of nodes in the WBAN. If there is only one DATA subsection configured in the insertion time slot, then the ACK frame can use the same structure as MEM-MAC’s ACK frame. If multiple DATA subsections are installed in the insertion time slot, the coordinator may need to acknowledge multiple nodes using the same ACK frame. For this, LDC-MAC assigns a node *N* in the WBAN a sequence number *i* and appoints the *i*-th bit in the data field to node *N*. The coordinator sets the *i*-th bit to be one to acknowledge node *N*. Then, if node *N* sends a frame to the coordinator using one DATA subsection, it will receive the ACK frame and check the *i*-th bit of the data field to verify the acknowledgment. Using this method, at least *n* bits should be installed in the data field of the ACK frame for a WBAN with *n* nodes. The coordinator can also exploit ACK frames to transmit control frames. For example, when the type field is “SYNC”, the data field carries the synchronization data, which can be exploited by the nodes to synchronize their clocks.

### 3.2. Operations of the MAC Protocol

In a WBAN, there are two communication directions, from the coordinator to the nodes and from the nodes to the coordinator. Most of the traffic is from the nodes to the coordinator, and only a minority of the traffic is from the coordinator to the nodes. We first introduce how LDC-MAC sends frames from the coordinator to the nodes, then how frames are transmitted from the nodes to the coordinator is introduced.

**Figure 6:**
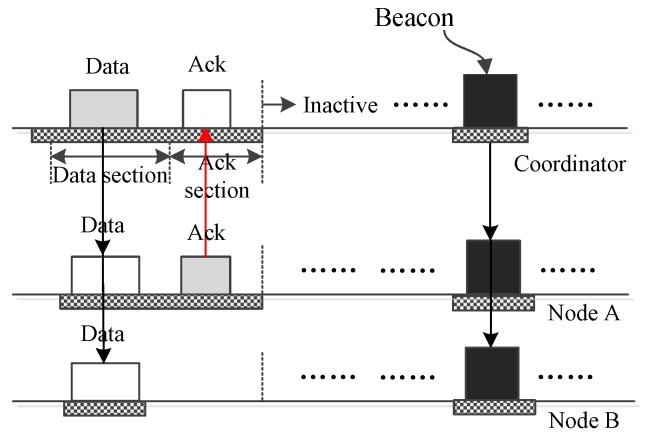
The coordinator sends small data to one node.

**Figure 7:**
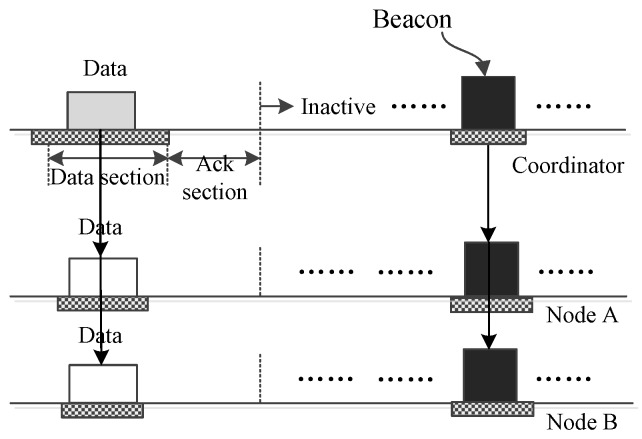
The coordinator broadcasts small data to all nodes.

How LDC-MAC transmits a small data frame or a control frame from the coordinator to one node is shown in Figure 6. The frame is transmitted using a download insertion time slot. Such an operation can be used to drive an actuator node. The data or command are encapsulated in the DATA subsection and broadcast to all nodes. Only the node with the right address accepts the frame and replies with an acknowledgement (ACK) frame. Other nodes just abandon the received frames. Figure 7 illustrates how the coordinator sends one small data frame or command frame to all nodes using a download insertion slot. Compared to Figure 6, the only difference is that the frame is not acknowledged. The above operation can also be accomplished using download insertion DATA subsections in bidirectional insertion slots, but in such a case, the coordinator cannot receive the acknowledgment even it only sends one frame to a node.

**Figure 8:**
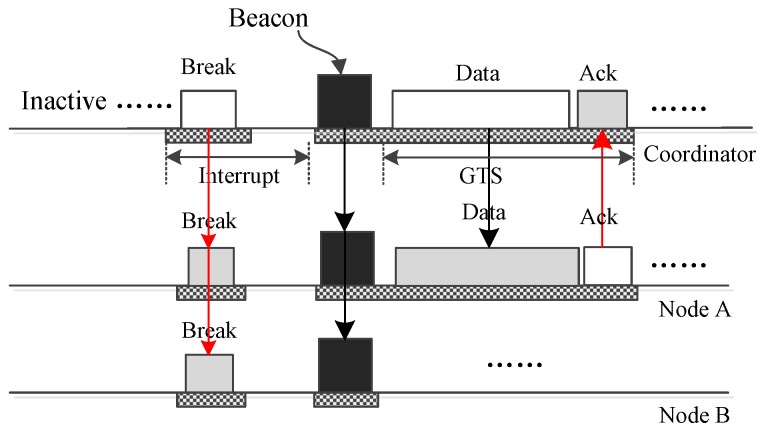
The coordinator sends big data to one node.

**Figure 9:**
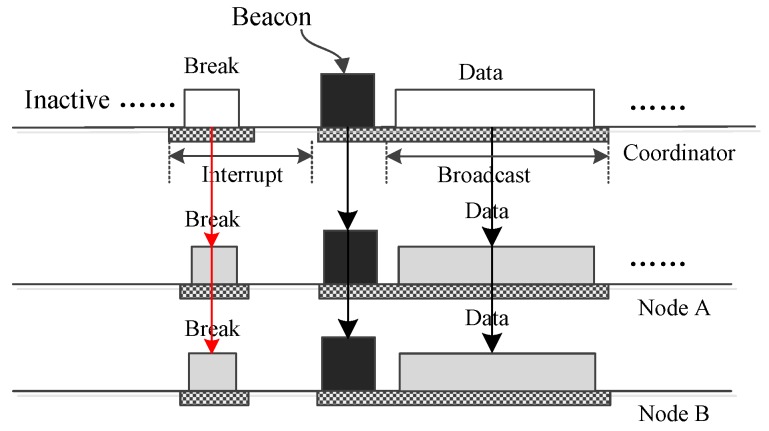
The coordinator broadcasts big data to all nodes.

The operations by which the coordinator transmits urgent big data to one or all nodes are displayed in Figure 8 and Figure 9, respectively. Here, these two operations are also completed using download insertion slots. To deliver the big data, one control frame is composed of the coordinator and sent to all nodes to end the ongoing superframe and commence a new one. Then, the big data are transmitted using a GTS slot or broadcast using a broadcast period. For unicast, the receiver node should acknowledge the data. For broadcast, there is no acknowledgment.

If the big data that the coordinator needs to transmit are not urgent, then it is not necessary to interrupt the running superframe immediately. The coordinator can wait till the end of the current superframe, and then, it can send the data in the new superframe. Besides the download insertion slots, the download DATA subsections of bidirectional insertion slots or the ACK sections of upload insertion time slots can also be adopted to interrupt the current superframe.

The upload operations are introduced next. According to the data size, data priority and number of frames to be sent, several cases may happen. We first introduce the case that there is no data to be transmitted. Next, the transmissions of small data and big data are introduced, respectively.

**Figure 10:**
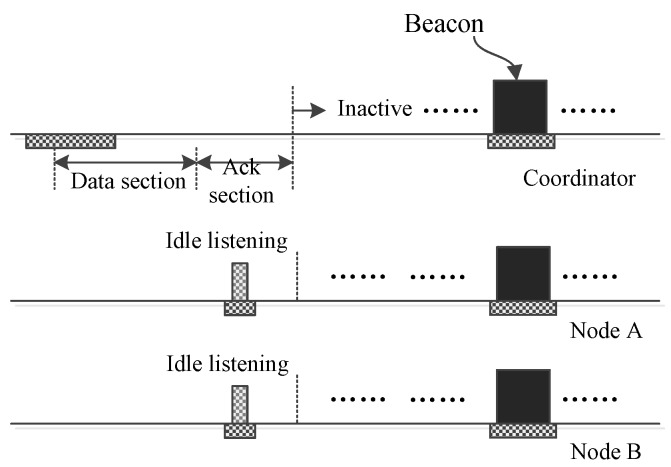
No node sends data to the coordinator.

In a WBAN in which all nodes are working in low data-rate monitoring mode, it is common that no event happens between two neighboring insertion time slots. Figure 10 illustrates how the nodes behave when there is no traffic. Because there are no data to send, all nodes sleep except the ACK section. Unlike the nodes, the coordinator needs to listen to the medium to sense possible frames. Once the coordinator judges that no transmission is going on, it abandons listening to save energy. Next, if the coordinator does not have a frame to send, it will fall into sleep to save energy. If the coordinator has a frame to send, it will broadcast the frame in the following ACK section. Figure 10 shows the case that the coordinator does not have a frame to send. In this case, each node will encounter a short idle listening. Then, it will perceive that there is not a frame being transmitted, and it will switch into sleeping mode.

**Figure 11:**
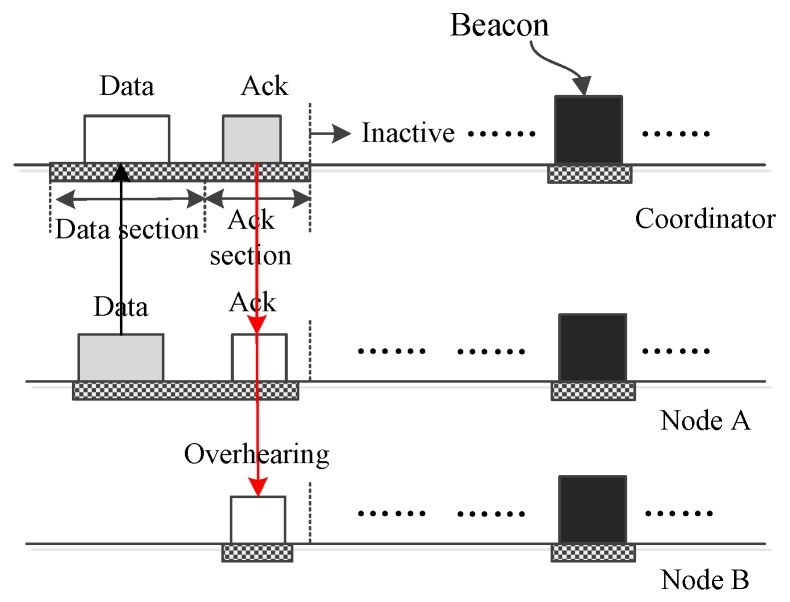
Only one node sends small data to the coordinator.

Figure 11 displays how a node *A* sends small data to the coordinator. As the figure displays, node *A* encapsulates the data into a frame and sends this to the coordinator in a DATA subsection. Then, the coordinator accepts the frame and acknowledges the frame. All nodes receive the ACK frame, but only node *A* accepts it, and other nodes just drop it.

**Figure 12:**
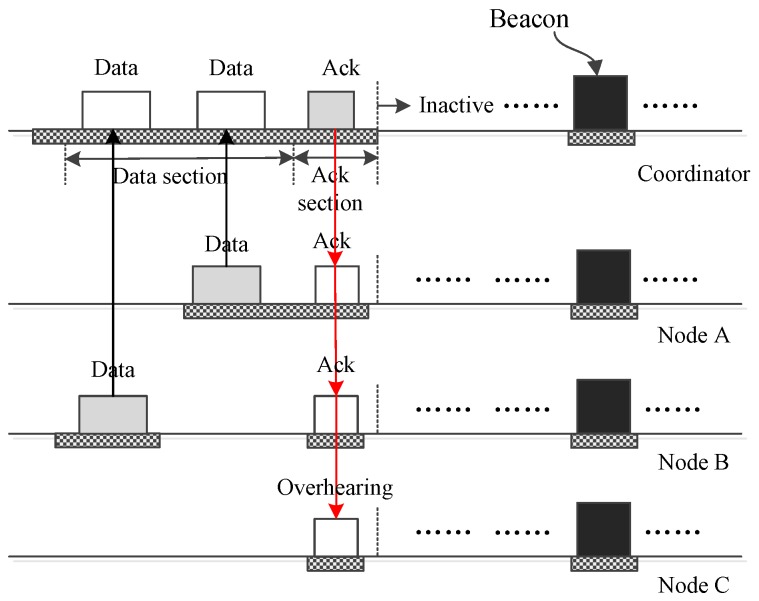
Two nodes send small data to the coordinator.

Figure 12 shows how two nodes transmit their frames to the coordinator using two different DATA subsections of one insertion time slot. If a WBAN contains dozens of nodes, it may be needed to allocate more than one subsection in the DATA section. By dividing the nodes to be the users of different subsections, it is effective to reduce the collision possibility. As the figure shows, node *A* and node *B* transmit their data in different subsections. Their data are acknowledged together by one ACK frame. As for the case that more nodes transmit their data using more DATA subsections, the process is similar to Figure 12.

**Figure 13:**
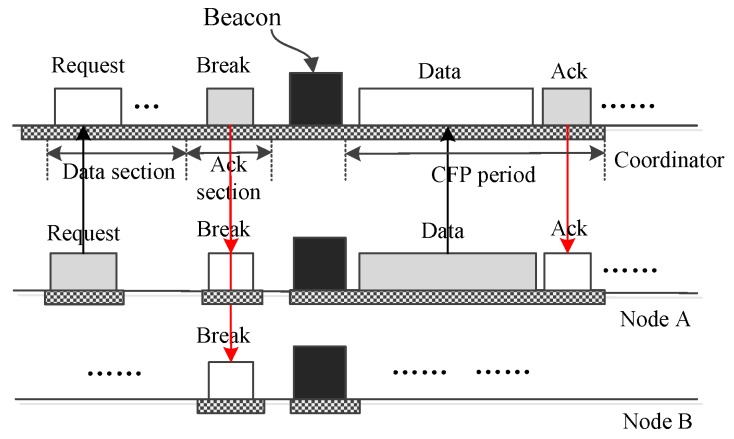
Only one node sends big data to the coordinator.

**Figure 14:**
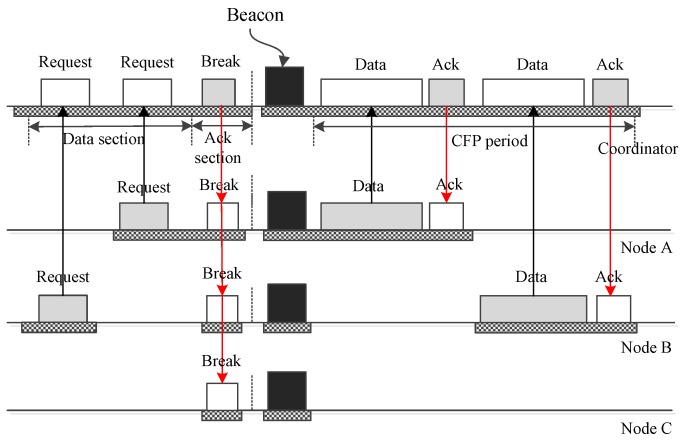
Two nodes send big data to the coordinator.

Next, we examine how a node uses LDC-MAC to transmit big urgent data. Figure 13 displays how a node *A* conveys big data. As the figure shows, a GTS request frame containing the data size and priority *p* is sent to the coordinator using a DATA subsection of an upload insertion slot. Upon receiving the request, the coordinator judges *p* to determine if it is necessary to break the current superframe immediately to deliver the big data. If *p* is not urgent enough, then the ongoing superframe is not interrupted, and the big data will be handled later. If *p* is urgent enough, then the ongoing superframe will be broken immediately by a control frame from the coordinator. In the new superframe, node *A* will use the GTS slots assigned to it to deliver its data. As for the cases for which there is more than one node having big data to send, Figure 14 gives a case illustrating how two nodes transmit their big data. As the figure reveals, nodes *A* and *B* send their requests using two DATA subsections, then they send their data to the coordinator using GTS slots in the new superframe.

**Figure 15:**
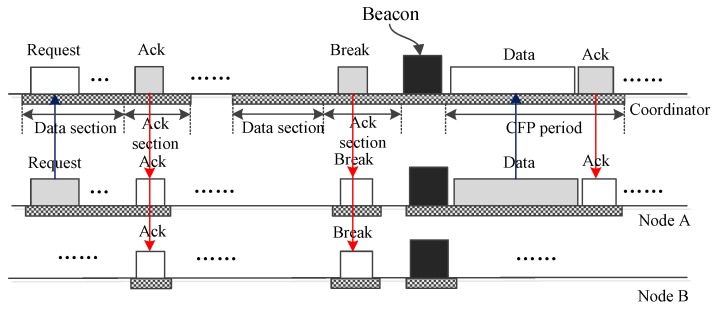
The case that a break is postponed.

As mentioned above, the break of a superframe may be postponed. Figure 15 shows such a case. As the figure shows, the ongoing superframe is not interrupted immediately after the coordinator receives the request because the coordinator has tasks of higher priority to handle. After the coordinator finishes the tasks, it composes and broadcasts a control frame to break the superframe. Then, in the next superframe, node *A* transmits its frame to the coordinator using the assigned GTS time slots.

**Figure 16:**
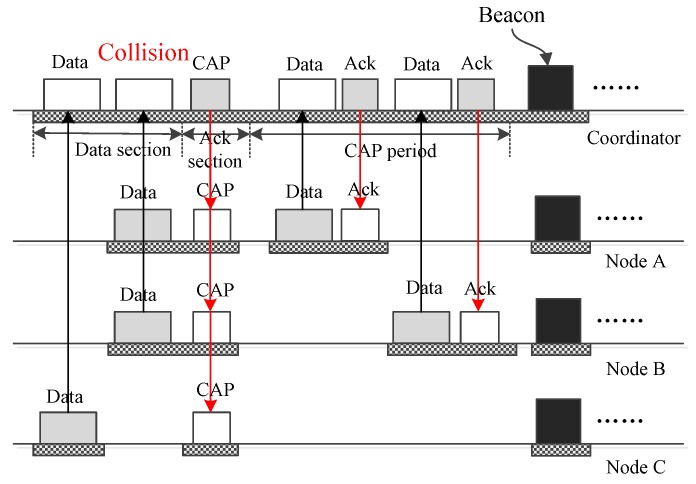
The case that a collision happens and CAP period is activated.

It is worth noting that collisions may happen when multiple nodes transmit their frames to the coordinator using the same DATA subsection. Figure 16 shows a case in which two frames from node *A* and node *B* collide with each other. As a result, the coordinator fails to receive the frames. The coordinator responds to this by composing a control frame and broadcasting it to all nodes to announce the introduction of a CAP period in which node *A* and node *B* can send their data frame or request frame to the coordinator. After the CAP period, the coordinator broadcasts a beacon frame to inform the nodes that a new superframe begins.

The above figures have illustrated the basic operations of LDC-MAC. More complicated traffic patterns can be transported by variants or combinations of the above basic operations. It has been mentioned above that LDC-MAC needs a mechanism to awaken the nodes rapidly when an emergent event is detected. Such a mechanism can be implemented by real-time broadcast of a network command from the coordinator to all nodes. In LDC-MAC, the DATA subsections of a download insertion slot, the download DATA subsections of a bidirectional insertion time slot and the download ACK sections can all be adopted to distribute such a command. Moreover, the coordinator can issue a control frame to interrupt the ongoing superframe, and then, in the following new superframe, more complicated commands can be issued to the nodes.

LDC-MAC is still suffering from overhearing and idle listening. Overhearing and idle listening only happen in the ACK subsections, download DATA subsections and broadcast period. An idle listening happens to a node when there is not a frame being transmitted during an ACK subsection, a download DATA subsection or a broadcast period. If there is a frame being transmitted, an overhearing occurs at a node *A* if the frame is not destined to *A*. By examining the channel or inspecting the content of the received packet, LDC-MAC can cast off overhearing and idle listening of a node to some extent. For the coordinator, the situations are different from the nodes. The coordinator does not have the overhearing problem, but it still is suffering from idle listening.

Last, but not least, all of the download sections of different kinds of insertion time slots could be adopted to send synchronization frames from the coordinator to each node. This is significant for LDC-MAC. The long superframe structure of LDC-MAC may introduce long clock drifts, which may cause the clocks of different nodes to lose synchronization. The out-sync may introduce energy waste and impair the communication. Hence, specific control frames are needed to resynchronize the clocks.

## 4. Performance Analysis

To analyze the performance of LDC-MAC, a WBAN with one coordinator *c* and *n* nodes is modeled as $WBAN=\left\{C,N_{1},N_{2},\ldots ,N_{n}\right\}$. Let *m* be the number of DATA subsections in an insertion slot, then the nodes can be divided to *m* groups correspondingly. Let $\left\{n_{1},n_{2},\ldots ,n_{m}\right\}$ be the set of node numbers of all groups, then $\sum_{i=1}^{m}n_{i}=n$. For the ease of analysis, let *n* be divisible by *m*, that is n=m×l, and *l* is a positive integer. For each node, let both occurrence processes of the small data and the big data obey the Poisson process. For a node $N_{i}$, let $\lambda_{iS}$ and $\lambda_{iB}$ be the average small data arrival rate and the average big data arrival rate. For all data arrival processes, each process is assumed to be independent from the other one. Then, according to the theory of a stochastic process, the small and big data arrival rates of node $N_{i}$ can be expressed as $\lambda_{i}=\lambda_{iB}+\lambda_{iS}$. For all nodes with average small data arrival rate set $\left\{\lambda_{1S},\lambda_{2S},\ldots ,\lambda_{nS}\right\}$ and average big data arrival rate set $\left\{\lambda_{1B},\lambda_{2B},\ldots ,\lambda_{nB}\right\}$, a new small data process with occurrence rate $\lambda_{S}=\sum_{i=1}^{n}\lambda_{iS}$ and a new big data process with occurrence rate $\lambda_{B}=\sum_{i=1}^{n}\lambda_{iB}$ can be drawn. Furthermore, a new all data process with occurrence rate $\lambda =\lambda_{B}+\lambda_{S}$ can be obtained by aggregating all of the data arrival processes.

For a WBAN using the LDC-MAC protocol, we assume here that the transceiver is switched into standby mode when it is not required in order to conserve energy. Because LDC-MAC is a beacon-enabled MAC protocol, we first calculate the duty cycle caused by beacon frames regularly transmitted and received. Suppose BI is the time interval between two adjacent beacon frames and $2\left(\varepsilon_{\mathrm{TX}}+\varepsilon_{RX}\right)\cdot BI$ is the clock drifts accumulated during BI; let $T_{Beacon}$, $T_{TX\_wu}$ denote the beacon frame transmission time, and let $T_{TX\_wu}$ and $T_{RX\_wu}$ be the transmitter’s switching time and the receiver’s switching time from standby mode to active mode; then the duty cycle incurred by the regularly-transmitted beacon frames can be expressed as [40]:

(1) $$DC_{Beacon}=\frac{2\left(\varepsilon_{\mathrm{TX}}+\varepsilon_{RX}\right)\cdot BI+T_{Beacon}+T_{TX\_wu}+T_{RX\_wu}}{BI}$$

From Equation (1), $DC_{Beacon}$ refers to the ratio of the time used for the reception of a regular beacon frame to a whole beacon interval.

Next, the energy power consumed by a node is calculated. The energy consumption of a node contains two parts, one for receiving operations and the other for transmitting operations. The energy power for receiving operations is analyzed first. In a low data-rate WBAN, because the nodes contribute far more traffic than the coordinator, only the upload operations are considered in the following analysis. The frames received by a node include the acknowledgment frames and potential broadcast frames transmitted in the ACK sections, the regular beacon frames and the adaptive beacon frames. As a result, the average receiving power consumption of a node can be calculated by:

(2) $$\begin{array}{cc} P_{Node\_RX} & =\left(DC_{Beacon}+\frac{T_{i\_ack}+T_{RX\_wu}}{T_{Event}}\right.\\ & \left.+\frac{{T_{i\_ack/}}_{2}+T_{RX\_wu}+{\bar{T}}_{i\_ack}+\left(1-P_{\left(x=1,S)\vee (x=0\right)}\right)\cdot \left({T_{i\_ack/}}_{2}+T_{Beacon}+T_{RX\_wu}\right)}{I_{Int}}\right)\cdot P_{RX} \end{array}$$

In Equation (2), $P_{RX}$ denotes the power cost by a node when its antenna is in receiving mode; $DC_{Beacon}$ is the duty cycle incurred by the regularly transmitted beacons; $T_{i\_ack}$ denotes the time length of an ACK section; $T_{Event}$ is the average data arrival interval; $\frac{T_{i\_ack}+T_{RX\_wu}}{T_{Event}}$ is the duty cycle incurred by the ACK frames; $\frac{{T_{i\_ack/}}_{2}+T_{RX\_wu}+{\bar{T}}_{i\_ack}+\left(1-P_{\left(x=1,S)\vee (x=0\right)}\right)\cdot \left({T_{i\_ack/}}_{2}+T_{Beacon}+T_{RX\_wu}\right)}{I_{Ins}}$ indicates the duty cycle incurred by the adaptive beacon frames and adaptive acknowledgment frames; $I_{Ins}$ signifies the insertion slot interval; and ${\bar{T}}_{i\_ack}$ denotes the adaptive ACK frames in the case that every node group only has no more than one small data packet. $P_{\left(x=1,S)\vee (x=0\right)}$ signifies the probability that every group has none or only one small data packet generated in the last insertion interval; therefore, $1-P_{\left(x=1,S)\vee (x=0\right)}$ means there is one group that has one big data packet or more than one data packet, and the ongoing superframe will be broken. $P_{\left(x=1,S)\vee (x=0\right)}$ can be given by:

(3) $$P_{\left(x=1,S)\vee (x=0\right)}=\prod_{i=1}^{m}\left(e^{-\left(\lambda_{i,B}+\lambda_{i,S}\right)\cdot I_{Ins}}+\lambda_{i,S}\cdot I_{Ins}\cdot e^{-\lambda_{i,S}\cdot I_{Ins}}\right)$$

where $\lambda_{i,B}$ and $\lambda_{i,S}$ denote the aggregated big data arrival rate and the aggregated small data arrival rate of group *i*, respectively.

The ${\bar{T}}_{i\_ack}$ in Equation (2) can be calculated by:

(4) $${\bar{T}}_{i\_ack}=\left(P_{\left(x=1,S)\vee (x=0\right)}\cdot \sum_{i=1}^{m}C\left(m,i\right)\cdot {\left(\frac{P_{x_{i}=1,S}}{P_{\left(x_{i}=1,S\right)\vee \left(x_{i}=0\right)}}\right)}^{i}\cdot {\left(\frac{P_{x_{i}=0}}{P_{\left(x_{i}=1,S\right)\vee \left(x_{i}=0\right)}}\right)}^{m-i}\cdot T_{i\_ack}\right)/2n$$

where $P_{x_{i}=1,S}$ and $P_{x_{i}=0}$ mean the probabilities that there is only one small data packet or there is no data generated in a group, while $P_{\left(x_{i}=1,S\right)\vee \left(x_{i}=0\right)}$ denotes the probability that there is no data or only one small data packet. For the ease of analysis, here it is assumed that every node has the same average small data arrival rate and average big data arrival rate. $P_{x_{i}=1,S}$, $P_{x_{i}=0}$ and $P_{\left(x_{i}=1,S\right)\vee \left(x_{i}=0\right)}$ can be calculated as in Equation (3).

Next, the energy power of a node for transmitting operations is analyzed. The transmitting operations of a node include the data transmissions and the additional data transmissions when collisions happen. Hence, the energy power consumed by data transmissions can be expressed as:

(5) $$\begin{array}{cc} P_{Node\_TX} & =\;(T_{Data}+T_{TX\_wu}+\left(1-P_{\left(x_{i}=1,s\right)\vee \left(x_{i}=0\right)}\right)\cdot \\ & \left(T_{TX\_wu}+T_{i\_data}+T_{CSMA}\right))\cdot P_{TX}/I_{Ins} \end{array}$$

where $T_{Data}$ is the DATA subsection time length, $T_{i\_data}$ denotes the data transmission time and $P_{TX}$ is the power cost by a node when its antenna is in transmitting mode. $T_{CSMA}$ is the average time for a node to compete for accessing the medium, and $T_{CSMA}$ can be calculated by [40]:

(6) $$T_{CSMA}=T_{TX\_wu}+R\cdot T_{CCA}+f\left(R,T_{BO(\alpha )}\right)$$

Next, the energy power consumed by the coordinator is calculated. The energy power for receiving operations is analyzed first. The receiving operations of the coordinator include receiving data frames and listening to the medium, except transmitting ACK frames to the nodes during the CFP and CAP periods. Hence, the power of the coordinator for frame receptions can be calculated by:

(7) $$P_{Coor\_RX}=\frac{T_{RX\_wu}+m\cdot T_{i\_data}+P_{Big}\cdot \left(T_{CFP}-\sum T_{Ack\_1}\right)+P_{x\geq 2}\cdot \left(T_{CAP}-\sum T_{Ack\_2}\right)}{I_{Ins}}\cdot P_{RX}$$

where $T_{CFP}$ is the length of the CFP period, $\sum T_{Ack\_1}$ is the total time cost by the ACK frames in the CFP period, $T_{CAP}$ is the length of the CAP period, $\sum T_{Ack\_1}$ is the the total time by the ACK frames in the CAP period, $P_{x\geq 2}$ is the probability that there is at least one big data packet and $P_{x\geq 2}$ signifies the possibility that there are at least two data packets. $P_{Big}$ and $P_{x\geq 2}$ can be calculated by:

(8) $$P_{Big}=\sum_{i=1}^{\infty }\frac{{\left(\lambda_{B}I_{Ins}\right)}^{i}}{i!}e^{-\lambda_{B}I_{Ins}}$$

(9) $$P_{x\geq 2}=1-P_{x\leq 1}=1-\prod_{i=1}^{m}\left(e^{-\lambda_{i}\cdot I_{Ins}}+\lambda \cdot I_{Ins}\cdot e^{-\lambda_{i}\cdot I_{Ins}}\right)$$

The transmitting operations of the coordinator contain broadcasting the regular and adaptive beacon frames and acknowledging the data frames. Therefore, the energy power cost on transmission is:

(10) $$\begin{array}{c} P_{Coor\_TX}=\left(DC_{Beacon}+\frac{T_{i\_ack}}{T_{Event}}+\right.\\ \left.\frac{\left(P_{x=1,B}+P_{x\geq 2}\right)\left(T_{Beacon}+T_{TX\_wu}\right)+P_{Big}\cdot \sum T_{Ack\_1}+P_{x\geq 2}\cdot \sum T_{Ack\_2}}{I_{Ins}}\right)P_{TX} \end{array}$$

Next, we analyze the frame delays of LDC-MAC for both small data and big data. Let $I_{Ins}$ be the insertion slot interval, then the waiting time of the data is $\frac{I_{Ins}}{2}$. For small data, these can be transmitted using one DATA subsection or using the CAP period; while big data are always transmitted using one CFP slot. Hence, the difference between the delay of small data and the delay of big data mainly lies in the different transmission methods. The average delay of a small data of node group *i* can be written as:

(11) $$D_{Small}=\frac{I_{Ins}}{2}+T_{Data}+T_{i\_ack}+\frac{P_{x_{i}\geq 2}\cdot T_{CAP}}{2(P_{x_{i}\geq 2}+P_{x_{i}=1})}$$

where $\frac{P_{x_{i}\geq 2}\cdot T_{CAP}}{2(P_{x_{i}\geq 2}+P_{x_{i}=1})}$ means if there is a collision, then it takes $\frac{T_{CAP}}{2}$ on average to transmit the data.

The average delay of a big data is calculated by:

(12) $$D_{Big}=\frac{I_{Ins}}{2}+\frac{T_{Ins}}{2}+T_{Data}+T_{i\_ack}+P_{x\geq 2}\cdot T_{CAP}+T_{Beacon}+\frac{T_{CFP}}{2}$$

where $T_{Ins}$ is the length of an insertion slot. Because big data cannot be transmitted using the insertion slots, the big data generated by one node *A* during the insertion slots has to wait for the DATA subsection of *A* to pass. Therefore, the waiting time of one big data packet has to include an extra $\frac{T_{Ins}}{2}$. The time lengths of $I_{Ins}$, $T_{CAP}$ and $T_{CFP}$ are all determined by the coordinator in the working time of LDC-MAC. By counting the the number of small data and big data and doing the statistical work, the coordinator can decide a proper $I_{Ins}$ and a proper $T_{CAP}$. $T_{CAP}$ is always set to be long enough, so that it can handle all potential frames. $T_{CFP}$ can be set in accordance with the CFP requests received by the coordinator during the CAP period.

Finally, we calculate LDC-MAC’s time slot utilization efficiency, which can be reflected by the ratio of the active time of both the coordinator and the nodes. This ratio can be written as:

(13) $$U_{LDC-MAC}=DC_{Beacon}+\frac{T_{Ins}+P_{x\geq 2}\cdot T_{CAP}+P_{Big}\cdot T_{CFP}}{I_{Ins}}$$

where $T_{Ins}$ is the length of the insertion time slot.

## 5. Performance Evaluation

A star WBAN with a coordinator and 24 nodes is adopted to evaluate the performance of LDC-MAC. The reason for using 24 nodes is that 24 can be divided exactly by many integers, e.g., 1, 2, 3, 4, 6, 8, 12 and 24. Hence, these 24 nodes can be divided using many group patterns. For each group pattern, all groups have the same number of nodes. OMNeT++ [41] is adopted as the simulation tool. The radio parameters are set according to CC2530 [42] which has been proposed by Texas Instruments as the second generation system-on-chip solution for 2.4 GHz IEEE 802.15.4. CC2530 has three power modes, Low Power Mode 2 (LPM2), Low Power Mode 1 (LPM1) and active mode (AM). LPM2 mode is the most energy-saving mode. We assume a node always switches its radio part into LPM2 when there is no transmitting or receiving task. To transmit or receive a frame, a node in LPM2 needs to wake up from LPM2 to AM mode and then to TX/RX modes. The mode transition timings and other parameters are listed in Table 1.

The performance of LDC-MAC is evaluated by comparing LDC-MAC with two other MAC protocols, 802.15.4 and MEM-MAC. The beacon length of 802.15.4 is assumed to be 30 bytes, while the beacon lengths of MEM-MAC and LDC-MAC are both set to be 34 bytes. For three MAC protocols, the lengths of an insertion DATA frame and an insertion ACK frame are set to be 10 bytes and six bytes. For LDC-MAC and MEM-MAC, the lengths of every DATA section and every ACK section are all configured to be 0.384 ms and 0.256 ms. These configurations are listed in Table 2.

The performance of 802.15.4 is closely related to the beacon interval, while the performances of MEM-MAC and LDC-MAC are closely related to their interposition/insertion intervals. We first examine how the performances of these MAC protocols change with these intervals. To do this, we fix the average data arrival speed and change these intervals and then examine how the performances change with these values. The average data arrival interval of every node is set to be 20 min, which is high enough to make sure that the impact caused by the low data-rate traffic can be ignored.

**Figure 17:**
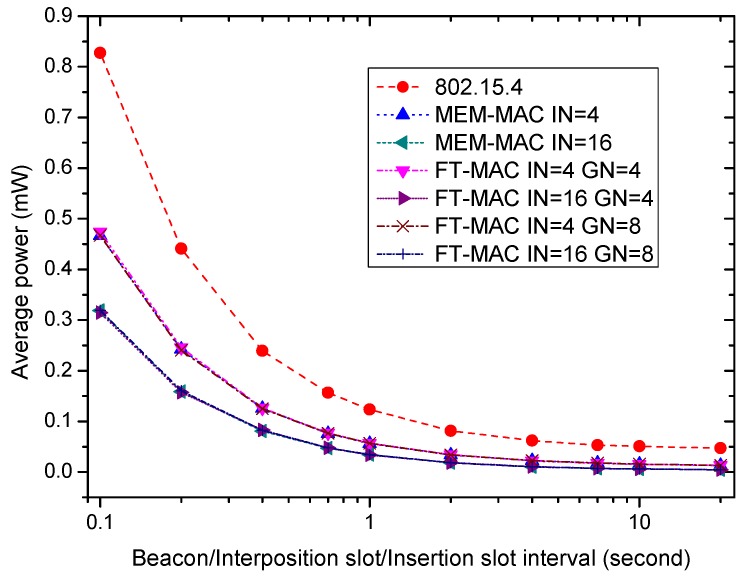
The effect of the beacon/interposition/insertion intervals on node average power.

Figure 17 displays how a node’s average energy power changes as the beacon/interposition/insertion intervals vary from low to high. Let $E_{allnodes}$ be the total energy consumption of 24 nodes in time period *T*, then a node’s average energy power $P_{node}$ is calculated by $P_{node}=\frac{E_{allnodes}}{24\times T}$. IEEE 802.15.4, two MEM-MAC schemes and four LDC-MAC schemes are compared. The MEM-MAC schemes and the LDC-MAC schemes are distinguished by how many interposition/insertion slots and node groups there are in a superframe. The number of interposition/insertion slots can be written as $IN=\frac{BI}{I_{Ins}}$, where BI represents the beacon interval and $I_{Ins}$ denotes the interposition/insertion slot interval. Two MEM-MAC schemes can be distinguished by IN. Let GN be the number of node groups used by LDC-MAC, then the LDC-MAC schemes can be differentiated by IN and GN. For an LDC-MAC scheme using GN, all nodes are partitioned to be GN groups, and each group is assigned one DATA subsection. Because the traffic is low, the energy for data transmission only contributes a very small part to the whole energy consumption. The power is mainly consumed by receiving the beacon frames and receiving packets during interposition/insertion slots. The figure shows that all of the curves decrease as the beacon/interposition/insertion intervals go up. This is caused by the reduction of beacon frames and interposition/insertion slots. Both MEM-MAC and LDC-MAC have higher energy efficiencies than 802.15.4, and this is obtained by the reduction of beacon frames. For an MEM-MAC scheme and an LDC-MAC scheme, even with different GN values, their average powers are basically the same if they uses the same IN. This shows that dividing nodes into groups is not energy efficient when the WBAN is under very low traffic.

**Figure 18:**
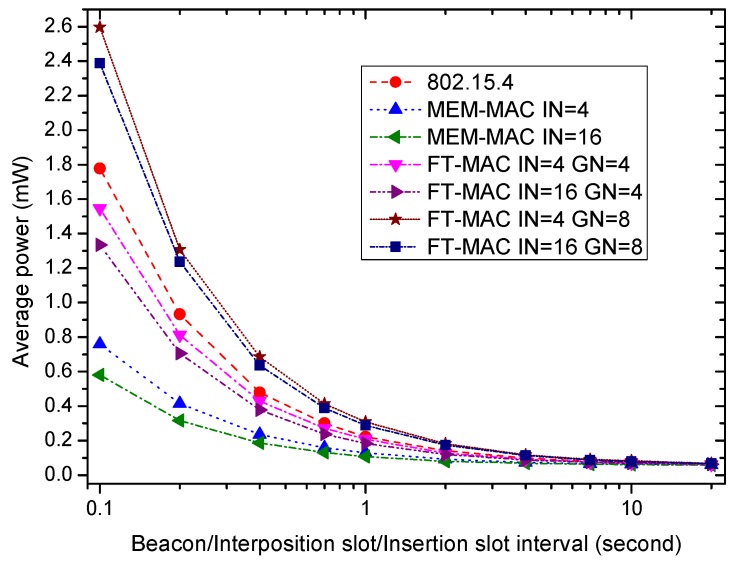
The effect of the beacon/interposition/insertion intervals on coordinator average power.

Figure 18 displays the effects of the beacon/interposition/insertion intervals on the average power of the coordinator. Let $E_{coordinator}$ be the total energy consumption of the coordinator in time period *T*, then the average power $P_{coordinator}$ can be calculated by $P_{coordinator}=\frac{E_{coordinator}}{T}$. The same seven MAC schemes used in Figure 17 are compared. The CAP period in a superframe of 802.15.4 is set to be 2 ms because the traffic is very sparse. Two GN values, four and eight, are used here. This means there are four or eight DATA subsections in an insertion slot of each LDC-MAC scheme. It can be seen that all of the average powers decrease as the beacon/interposition/insertion intervals increase. The energy consumed by 802.15.4 is mainly for transmitting the beacon frames. Compared to 802.15.4, the two MEM-MAC schemes reduce their power by cutting off the amount of the beacon frames. The LDC-MAC schemes can also lower the energy consumed on beacon frames, while they add new expenses by using additional DATA subsections. The new expenses increase as the GN value goes up. The power of two LDC-MAC schemes with GN = 4 is less than 802.15.4, while the power of two LDC-MAC schemes with GN = 8 is bigger than 802.15.4. This shows that the energy consumption of one LDC-MAC scheme when GN takes a big value is considerable.

**Figure 19:**
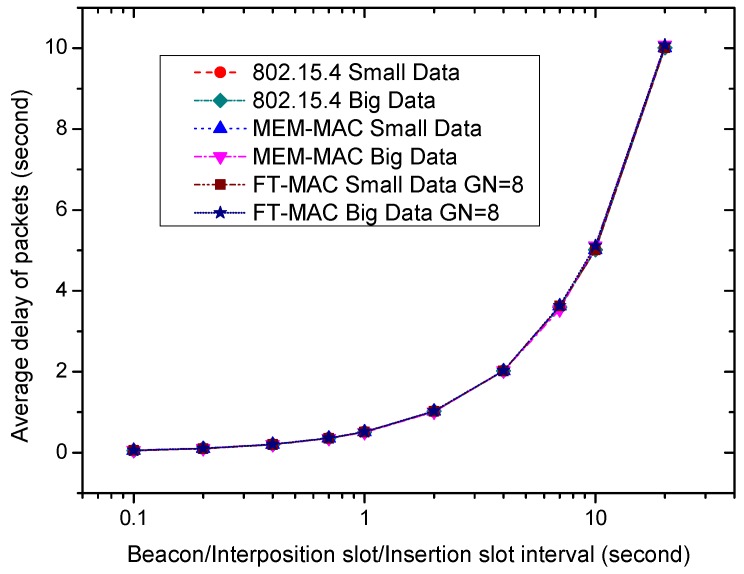
The effect of the beacon/interposition/insertion intervals on average frame delay.

**Figure 20:**
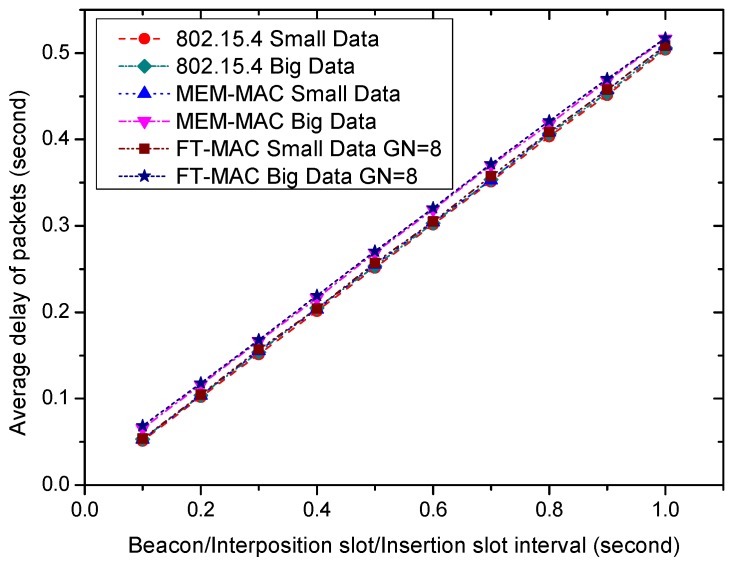
The enlarged part of Figure 17 from 0.1 s to 1.0 s.

It is revealed by Figure 17 and Figure 18 that longer beacon/interposition/insertion intervals can lower the energy consumption of a node. The disadvantage of longer beacon/interposition/insertion intervals can be reflected by longer data transportation delays. Figure 19 illustrates the effect of the beacon/interposition/insertion intervals on the average frame delays. As the figure reveals, longer beacon/interposition/insertion intervals always incur longer data delivery delays by lengthening the waiting time of the frames. Figure 19 shows that the delays are very close, and this shows that the MAC schemes can all transmit the data on time when the traffic is very low. It is hard to distinguish the differences between these delays. To observe these delays more clearly, the part from 0.1 s to 1.0 s of Figure 19 is enlarged and redrawn in Figure 20. The curves are still close, yet it can be observed that the data delays, especially the big data delays of MEM-MAC and LDC-MAC, are a little bigger than 802.15.4. The extra delays caused by the adaptive beacon frames and the CFP periods for transmitting the bigger data are responsible for the longer big data delays. It is worth noting the GN value of the LDC-MAC scheme in Figure 19 is eight. For an LDC-MAC scheme with a higher GN value, the delay of the big data is even higher. The longer waiting time introduced by the longer insertion slot is responsible for this.

Using the same WBAN, next, we fix the beacon/interposition/insertion intervals to be 0.5 s and investigate how the data arrival interval affects the performances of the MAC protocols. Six MAC schemes are used in the following simulations. One is 802.15.4; another is MEM-MAC; and the others are four LDC-MAC schemes whose GN values are set to be 3, 6, 12 and 24. This means each node group in the WBAN has 8, 4, 2 or 1 nodes for four different LDC-MAC schemes. The IN values of the MEM-MAC and four LDC-MAC schemes are all set to be four. It is worth noting that every node is assigned a DATA subsection when GN = 24, and this is actually a kind of TDMA MAC scheme. For the sake of simplicity, the average data arrival intervals of all nodes are set to be identical. Ten percent of the data are set to be big data, and the other data are small data. The size of one big data frame is set to be 40 bytes.

**Figure 21:**
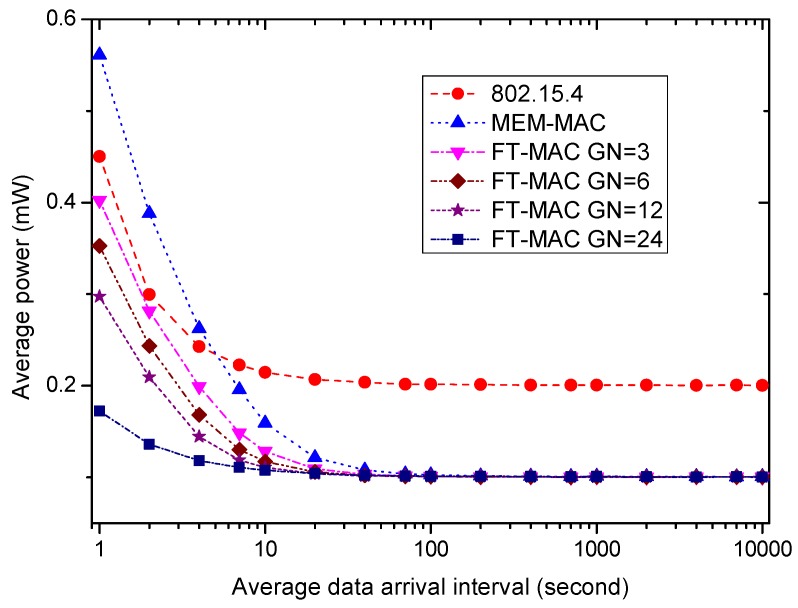
The effect of the average data arrival interval on the node average power.

Figure 21 exhibits how a node’s average energy consumption varies as the average data arrival interval varies from 1 s to 10,000 s. It is easy to compute that the amount of data occurring during a beacon/interposition/insertion interval changes from 12 to 0.0012. From the figure, the MEM-MAC scheme and four LDC-MAC schemes consume lower energy than 802.15.4 when the traffic is low, i.e., the average data arrival interval is bigger than 10 s. As explained above, the energy savings are mainly contributed by the reduction of beacon frames. As the average data arrival interval decreases, the MEM-MAC’s average power grows very quickly, and it exceeds 802.15.4 at 6 s at which there are two data packets that can be generated in one beacon/interposition/insertion interval on average. As the data interval is 1 s, MEM-MAC’s average power is much bigger than 802.15.4. The active beacon frames, CAP1 and CAP2 periods caused by frame collisions are responsible for this swift growth. The average powers of four LDC-MAC schemes also increase as the traffic grows, yet their incremental speeds are lower than 802.15.4. This is realized by using multiple DATA subsections to alleviate the frame collisions and using the CFP phase to convey the big data.

**Figure 22:**
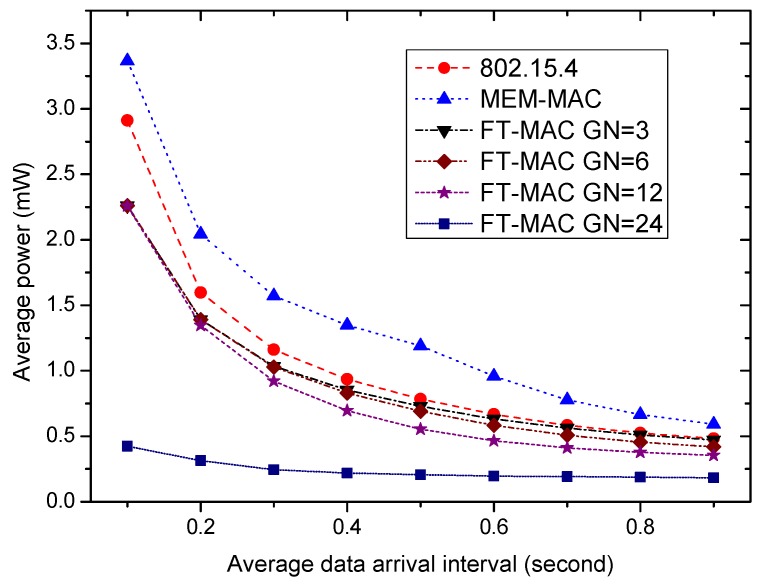
The effectiveness of using multiple DATA subsections and using the CFP period.

To verify the effectiveness of using multiple DATA subsections and using the CFP period further, more simulations were carried out for cases with denser traffic, and the results are presented by Figure 22. The average data arrival interval changes from 0.1 s to 0.9 s, and the average number of frames generated in one beacon/interposition/insertion slot changes from 120 to 13.3. To convey so many data frames in an energy-efficient way is a big challenge for the MAC protocols. For a node with multiple frames, transmitting the frames one by one is too inefficient. To deliver the data more efficiently, multiple data frames are aggregated to be a big data frame if one node has multiple data frames. It can be seen from the figure that the energy efficiency of MEM-MAC is always worse than 802.15.4 as the traffic is busy, and the four LDC-MAC schemes can manage to maintain their energy efficiencies better than 802.15.4. The energy efficiency of the LDC-MAC GN = 24 scheme is very prominent. As mentioned above, the LDC-MAC GN = 24 scheme is actually a TDMA scheme, and this ensures the high energy efficiency by letting a node only wake up in its own time slots and to sleep in other time slots.

**Figure 23:**
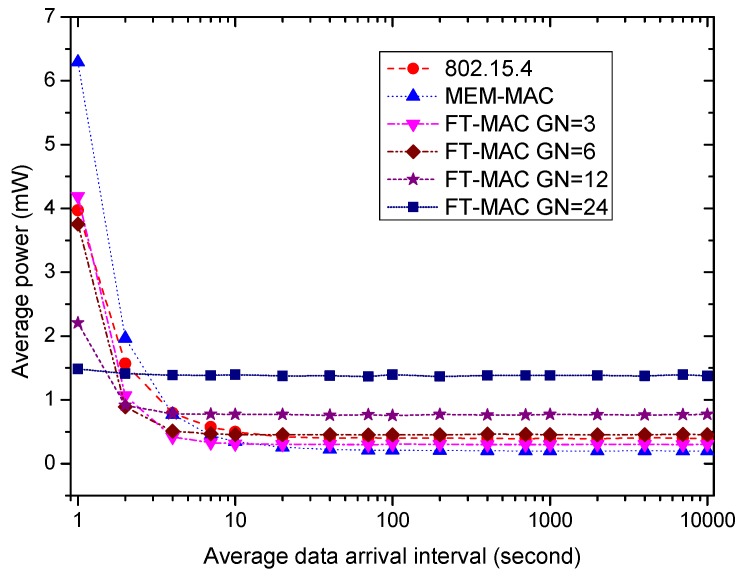
The effect of the average data arrival interval on coordinator average power.

Figure 23 displays the effect of the average data arrival interval on the coordinator’s average power. As the traffic is low, i.e., the average data arrival interval is bigger than 10 s, the average powers from high to low are LDC-MAC GN = 24, LDC-MAC GN = 12, LDC-MAC GN = 6, LDC-MAC GN = 3, 802.15.4 and MEM-MAC. This is because MEM-MAC only uses one DATA subsection, while the LDC-MAC schemes use multiple DATA subsections in one interposition/insertion slot. As the traffic grows, the coordinator needs to increase its duty cycle to convey more traffic, e.g., the CAP periods should be activated, and enough lengths should be allocated so as to deliver all of the data frames. Therefore, the power of all MAC schemes increases while the traffic becomes high. When the data interval decreases to 1 s, the average power from high to low changes to MEM-MAC, LDC-MAC GN = 3, 802.15.4, LDC-MAC GN = 6, LDC-MAC GN = 12 and LDC-MAC GN = 24. Because MEM-MAC only uses one DATA subsection, the frame collisions become very frequent as the traffic becomes high. The highest coordinator energy consumption of MEM-MAC is contributed by the high duty cycle caused by the adaptive beacons and two CAP periods. By using multiple DATA subsections and replacing the CAP2 period with CFP periods, the LDC-MAC schemes can achieve higher coordinator energy efficiencies than MEM-MAC.

**Figure 24:**
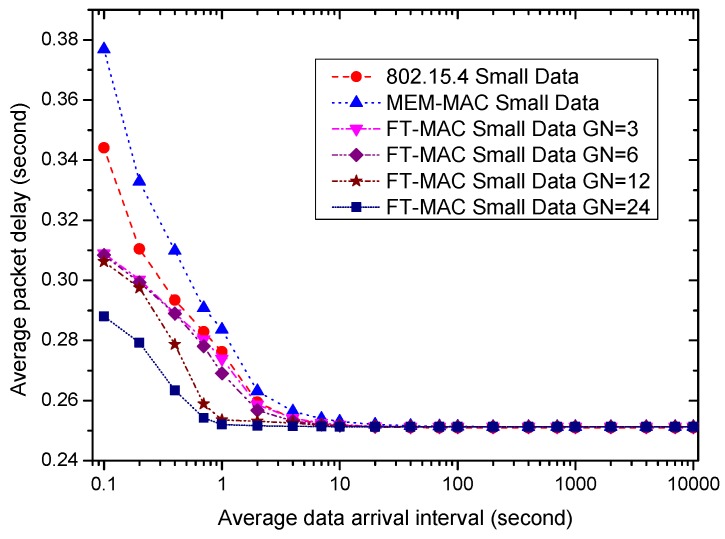
The effect of the average data arrival interval on average small data delay.

**Figure 25:**
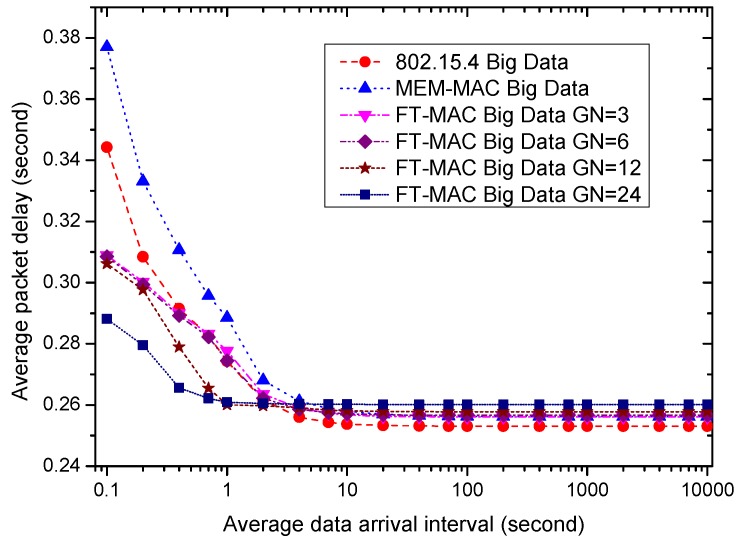
The effect of the average data arrival interval on average big data delay.

Next, the average data delivery delays of all MAC schemes are examined. Figure 24 and Figure 25 display the average small data delays and the average big data delays, respectively. As the figures show, all delays increase as the data interval decreases. The small data delays are very close when the traffic is sparse, e.g., the average data arrival interval is bigger than 10 s. Different from the small data delays, the big data delays of all of the MAC schemes are different when the traffic is sparse. As the traffic is rather low, the big data delays from large to small are LDC-MAC GN = 24, LDC-MAC GN = 12, LDC-MAC GN = 6, LDC-MAC GN = 3, MEM-MAC and 802.15.4. This can be explained by the adaptive beacons and insertion slots that increase the waiting time. All of the delays, including all big data delays and all small data delays, become bigger as the traffic becomes high. The increasing speeds of the small data delays as the traffic increases from high to low are MEM-MAC, 802.15.4, LDC-MAC GN = 3, LDC-MAC GN = 6, LDC-MAC GN = 12 and LDC-MAC GN = 24. The increasing speeds of the big data delays are similar to the small data delays. The reason to explain this is that MEM-MAC uses two CAP periods, and LDC-MAC schemes integrate CSMA and TDMA methods. As the traffic is high, e.g., the average data arrival interval is smaller than 0.5 s, the small data delays from high to low are MEM-MAC, 802.15.4, LDC-MAC GN = 3, LDC-MAC GN = 6, LDC-MAC GN = 12 and LDC-MAC GN = 24, and the big data delays exhibit the same sorting sequence. Actually, for every MAC scheme, the big data delay is very close to the small data delay when the the average data arrival interval is smaller than 0.5 s. This is because both the big data and the small data are aggregated into the same frame and transmitted together when a node has both big data and small data to send.

**Figure 26:**
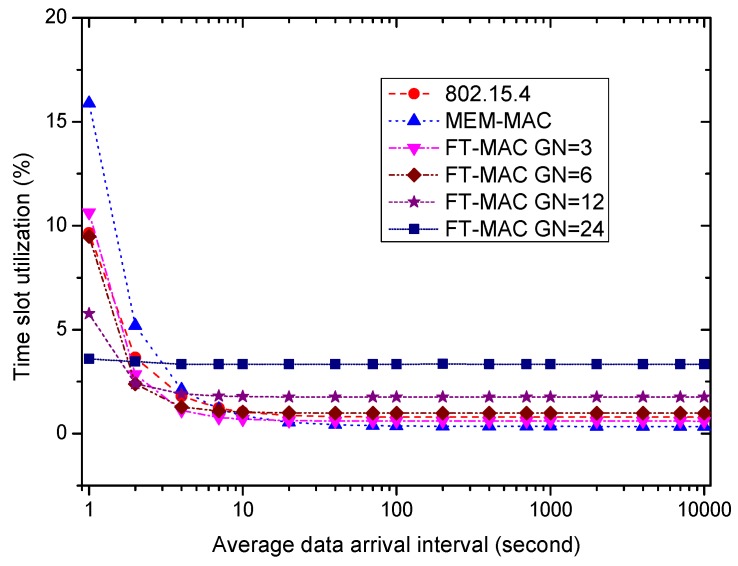
The effect of the average data arrival interval on time slot utilization.

Finally, how the time slot utilizations change with the average data arrival interval is examined. As Figure 26 displays, the time slot utilizations from high to low are LDC-MAC GN = 24, LDC-MAC GN = 12, LDC-MAC GN = 6, LDC-MAC GN = 3, 802.15.4, and MEM-MAC as the traffic is rather low, e.g., the average data arrival interval is bigger than 10 s. The reason for this is that MEM-MAC only uses one DATA subsection, while the LDC-MAC schemes use multiple DATA subsections in one interposition/insertion slot. As the traffic increases, the time slot utilization of every MAC scheme needs to be increased to accommodate more traffic. As a result, the time slot utilizations of all MAC schemes become higher as the traffic grows. The orders of the time slot utilizations change are MEM-MAC, LDC-MAC GN = 3, 802.15.4, LDC-MAC GN = 6, LDC-MAC GN = 12 and LDC-MAC GN = 24, as the data interval is 1 s. It can be observed the the change rule of the time slot utilizations accords with the change rule of the coordinator average power revealed by Figure 21. Similarly, this change rule can also be explained by the change rule of the coordinator average power.

To sum up, LDC-MAC can achieve higher energy efficiency by reducing the number of beacon frames. As the data arrival frequency is very low, e.g., the average data arrival interval is larger than 40 s, the average node energy consumption of 802.15.4 is the lowest, and the average node energy consumption of LDC-MAC is basically equal to MEM-MAC. As the average data arrival interval reduces, the node energy efficiency of MEM-MAC deteriorates more quickly than the other MAC schemes. By using additional DATA subsections and a CFP period, the energy efficiencies of the LDC-MAC schemes deteriorate more slowly than 802.15.4. As for the coordinator side, as the traffic is very low, MEM-MAC owns the best energy efficiency. LDC-MAC GN = 6, LDC-MAC GN = 12 and LDC-MAC GN = 24 have poorer energy efficiencies than 802.15.4 because they use considerable DATA subsections. As the traffic grows dense, the coordinator energy efficiency of MEM-MAC also deteriorates more quickly than the other MAC schemes. The small data delays of all MAC schemes are very close when the traffic is very low, while the big data delays of the LDC-MAC schemes are bigger than MEM-MAC and 802.15.4. While the average data arrival interval reduces, all of the small data and big data delays increase. As the traffic is busy enough, e.g., the data arrival interval is smaller than 0.5 s, the small data delay and the big data delay of every MAC scheme are close.

Based on the above summary, MEM-MAC should be adopted when the traffic is very low, e.g., the average data arrival interval is above 40 s. This shows that MEM-MAC works well for a medical emergency monitoring WBAN in which only very sparse traffic is generated most of the time. As the traffic becomes denser, e.g., the average data arrival interval is between 1 s and 40 s, LDC-MAC GN = 3, LDC-MAC GN = 6, LDC-MAC GN = 12 or other LDC-MAC schemes with different GN values can be adopted considering the node and coordinator energy requirements and the delay requirements. While the traffic becomes even denser, e.g., the average data arrival interval is below 1 s, LDC-MAC GN = 24 is the best choice. This also to some extent proves that TDMA is more efficient than CSMA when the traffic is busy.

## 6. Conclusions

In this paper, the medical monitoring task and two monitoring modes of a WBAN node are analyzed. To extend its lifetime, a node should try to stay in low data-rate monitoring mode as much as possible. Focusing on the requirements of the nodes working in low data-rate monitoring mode, a beacon-enabled adaptive MAC protocol (LDC-MAC) is proposed. Longer superframes are used by LDC-MAC to cut down the overhead of redundant beacons to save energy. Short insertion time slots embedded in the inactive periods are used to provide opportunities for low data-rate monitoring nodes to transmit their data. For a WBAN of dozens of nodes and busier data-rate monitoring traffic, multiple DATA subsections can be used as a makeshift means to alleviate the collisions. This mechanism integrates CSMA and TDMA together and can support complicated traffic modes. By adopting this flexible structure, LDC-MAC can support a WBAN in which low data-rate monitoring nodes and high data-rate monitoring nodes coexist. Simulation results show that LDC-MAC performs better than MEM-MAC under the condition of busier low data-rate monitoring traffic.

Low data-rate monitoring traffic produced by a WBAN may vary greatly with time. With the ability to change the number of DATA subsections in an insertion time slot, LDC-MAC has the potential to adapt to the time-varying low data-rate monitoring traffic. We will research this issue next. This includes optimizing the number of the DATA subsections in an insertion time slot, optimizing the length of the CAP period used under certain low data-rate monitoring traffic and designing the adaption algorithm.

## Figures and Tables

**Table 1:** Radio parameters. LPM, low power mode; AM, active mode.

Parameter	Value
LPM2 → AM time	0.3 ms
LPM1 → AM time	0.2 ms
AM → RX/TX time	192 μs
RX/TX turnaround time	192 μs
TX/RX turnaround time	192 μs
Voltage supply	1.8 V
Receive current	22.3 mA
Transmit current (0 dBm)	25.8 mA
AM current	1.6 mA
LPM1 current	175 μA
LPM2 current	30 nA

**Table 2:** Parameters related to MAC protocols.

Parameter	Value
Bit rate	250 kbps
802.15.4 beacon	0.96 ms
LDC-MAC/MEM-MAC beacon frame	1.088 ms
Small data frame	0.32 ms
Big data frame	1.6 ms
Acknowledgment frame	0.192 ms
LDC-MAC Insertion DATA subsection	0.384 ms
LDC-MAC Insertion ACK section	0.256 ms
MEM-MAC Interposition DATA section	0.384 ms
MEM-MAC Interposition ACK section	0.256 ms
Clock drift rate	30 ppm
CCA time	0.128 ms
